# The Relationship Between Personality Traits, Resilience, School Support, and Creative Teaching in Higher School Physical Education Teachers

**DOI:** 10.3389/fpsyg.2020.568906

**Published:** 2020-09-24

**Authors:** Qian Deng, Bing Zheng, Jing Chen

**Affiliations:** ^1^School of Sports Science and Physical Education, Southwestern University of Finance and Economics, Chengdu, China; ^2^Department of Physical Education, Tangshan Normal University, Tangshan, China; ^3^Department of Basic Education, Sichuan College of Architectural Technology, Deyang, China

**Keywords:** personality traits, resilience, school support, creative teaching, mediating effect

## Abstract

The investigation has been carried out on the *status quo* of higher school physical education teachers’ personality traits, resilience, and creative teaching status. The exploratory and confirmatory factor analyses, combined with multiple stratified linear regression analysis, were used to verify the data obtained by the structure model. The results show that (1) among the big five personality traits, excepting conscientiousness, the rest of the four dimension personality traits have different influences on creative teaching; (2) extraversion, agreeableness, and openness can produce an intermediary effect on innovative teaching through different dimensions of resilience; (3) the school support has a positive influence on five aspects of the creative teaching; and (4) problem cognition and empathy in resilience play a multilevel role of mediating effect and are regulated variables as well. The findings of the present study revealed that the key to success to creative teaching is to understand teachers’ personality traits, pay attention to the resilience of the development of teachers’ creative teaching, and provide required support; the higher the awareness of the problem and the degree of school support in the resilience was, the higher the problem solving and the higher degree of teaching in the creative teaching tended to be.

## Introduction

In the era of mass entrepreneurship and innovation, classroom teaching in school is facing enormous challenges. In addition to basic teaching tasks, it is necessary to adopt a variety of teaching designs to cultivate students’ innovative awareness and thereby to change the stiff and inflexible traditional teaching model (Gu, 2018; Huang et al., 2019a). Creativity is a crucial element of education (Liu and Chang, 2017), From the definition of creativity, some scholars (Soh, 2000; Beaird et al., 2018) pointed out that creative teaching is that teachers conceive, design, and use novel teaching orientations, methods, or activities to adapt to students’ mental development and stimulate students’ motivation to learn, so as to obtain the best teaching effect. Other scholars (Ozkal, 2014; Gu, 2018; Huang et al., 2019b) believed that creative teaching is the strategies to use a variety of novel and valuable teaching under the guidance of certain teaching thoughts to enhance students’ learning interest and motivation and to achieve teaching goals. The key to creative teaching is to develop and use novel, original, or inventive teaching methods (Khurshid et al., 2012). Relevant research shows that the higher the intrinsic motivation of teachers’ creative teaching, the more innovative is performance in teaching (Ozkal, 2014; Yalcin and Kilic, 2014).

Personality traits are an inherent tendency, representing the uniqueness of each individual, and have a sustained and stable influence on individual behavior and thoughts (Satchell et al., 2017). Empirical research shows that of all the variables that affect creativity, personal factors are the most explanatory, and the creative people often possess certain specific personality traits (Fajkowska and DeYoung, 2015; Huang et al., 2019a). Frith et al. (2019) and other scholars have pointed out that teachers with excellent scientific competition achievements have many personality traits and abilities, such as broad interests, away from prejudice and outdated methods, and creativity-related cognitive skills, which are conducive to innovation. Rubenstein et al. (2018) reported that factors affecting teachers’ creative teaching in the field of integrated learning include types of thinking, personality traits, family factors, growth and educational history, teaching beliefs, motivation, personal effort, productive professional skills, and pleasant organizational environment. Therefore, understanding the influence of personality traits of physical education teachers on their creative teaching has become the first exploration goal in the present study.

The resilience comes from the field of psychology, which was initially used to describe children’s experiences of being exposed to distress (such as abuse, trauma, and parental divorce), and eventually can develop positive results (Gu and Day, 2007; Mansfield et al., 2012; Yu and To, 2019). Based on this, resilience is regarded as an important personal asset that regenerates from frustration and rebounds in adversity. Its definition has three orientations: (1) regarding resilience as an individual ability; (2) regarding resilience as a process of adaptation; and (3) regarding resilience as a result of adaptation. Teachers’ resilience means that teachers can adapt to various situations through adjustment and enhance their ability to face adverse conditions. It can promote teachers to maintain their commitment to teaching and adopt specific strategies promptly (Castro et al., 2010; Chao et al., 2017). Villasana et al. (2017) further decomposed teachers’ resilience into “dangerous factors” and “protective factors,” of which protective factors were further divided into “internal” and “external” aspects. Internal protective factors include personal skills and orientation, such as social ability, problem-solving ability, proficiency, autonomy, and the ability to perceive the goal and the future; external protective factors include three aspects of families, schools, and communities, such as family support, opportunities to social intercourse, positive learning experience, and care for teachers (Soh, 2000; Campbell-Sills et al., 2006). Teachers with better resilience, when they encounter difficulties or difficulties in the teaching process, can often quickly adjust the balance between situational needs and behavioral responses and quickly recover from many challenges and frustrations they face (Masten, 2011; Zimmerman et al., 2013; Beaird et al., 2018). Positive psychology refers to when people encounter challenges or setbacks; they will have the ambition to solve problems and continuously practice to change their thinking, strengthen the positive force, and meet the challenges (Liu and Chang, 2017). Therefore, to explore whether teachers’ resilience has an intermediary effect between the five personality traits and creative teaching, to find out the mechanism to stimulate the innovative teaching has become the second exploration goal in the present study.

Resilience plays an essential role in the creative performance of teachers, and physical education is quite different from other education courses (Mancini and Bonanno, 2009). It focuses on physical activities and has the characteristics of diversity, spatialization, and humanization; in addition to developing a person’s motor skills and mental intelligence, physical education teaching often uses collective activities to promote positive personality and social development of youth. Therefore, in the environment of physical education teachers, if school support can provide more help for teachers’ resilience, it may be more conducive to promoting the occurrence of creative performance; conversely, if environmental factors damage teachers’ resilience, their creative teaching performance may be reduced. Therefore, school ecological factors may directly affect the creativity of teachers, or they may indirectly affect the creative performance of individuals by stimulating or inhibiting the resilience of individual creativity. That is, resilience may have an intermediary effect between school support and creative teaching performance. This has also become the third exploration goal that this study wants to explore.

However, in the past decade, domestic scholars’ research on the creative teaching of physical education teachers has remained at a single level. Few studies have discussed the impact of different factors on the creative teaching of physical education teachers from a multilevel perspective. However, the research on the relationship between physical education teachers’ personality traits, resilience, school support, and creative teaching is even blank. In this study, when discussing the impact of physical education teachers’ personality traits on creative teaching, we considered the mediating effect of school support and resilience. Based on this, three hypotheses were proposed: (1) big five personality traits of physical education teachers’ can positively affect creative teaching behavior; (2) the big five personality traits of physical education teachers can indirectly affect creative teaching behavior through the intermediary role of resilience, and (3) school support can directly affect creative teaching behavior and indirectly influence creative teaching behavior through the intermediary role of resilience.

## Subjects and Methods

### Subjects

The investigation of the cross-sectional study was conducted on public college and university physical education teachers in Chengdu and Chongqing using random sampling. Eighteen college and universities were selected to carry out this investigation, and 450 questionnaires were sent out, 443 were recovered, 7 invalid questionnaires were removed, and 436 valid questionnaires remained. Among them, 266 were males (60.0%) and 170 females (40.0%). The age range was from 28 to 63 years (*M* = 42.5, *SD* = 12.7). The average age for the male was 44.7 years (*SD* = 16.3); for the female, 40.3 years (*SD* = 11.4).

## Methods

### Measurement Instruments

#### The personality traits rating scale

The personality trait rating scale compiled by Witt et al. (2009) includes s five personality trait classification framework consisting of five constructs of 32 items with positive and negative questions. The following five constructs are Trait A: Agreeableness, which refers to a personality trait that is easy to get along with, to communicate, and to cooperate with (8 items); Trait B: Conscientiousness, which refers to a person’s concentration on the pursuit of goals (6 items); Trait C: Extroversion, which refers to the degree to which a person is comfortable with the relationship with others (6 items); Trait D: Neuroticism, which refers to the number and intensity required to stimulate a person’s negative emotional stimulation (7 items); and Trait E: Openness: which refers to the degree of absorption of facts and novelty (5 items).

#### The resilience rating scale

The resilience rating scale is adapted from teachers’ resilience rating scale by Wang et al. (2019). There are four constructs of 27 items, namely, problem cognition (10 items), hope optimism (6 items), empathy (8 items), and emotion regulation (3 items).

#### The school support rating scale

The school support rating scale is adapted from the organization support rating scale by Chen et al. (2014), which contains five items, including “The school emphasizes my values,” “The school cares about my well-being,” “The school will lend a helping hand when I need special assistance,” “The school takes pride in my work achievements,” and “The school emphasizes my contribution.”

#### The creative teaching rating scale

This creative teaching rating scale compiled by Wei et al. (2015) was adopted, which is mainly used to evaluate the innovative behavior of teachers in teaching. It contains 20 items and consists of five constructs: interactive discussion (3 items), open mind (3 items), problem-solving (5 items), multiple-level teaching (3 items), and independent learning (6 items).

### Procedures

This study was approved from the Southwest University of Finance and Economics.

By telephone and email, the researchers in this study requested a teacher each school in charge of the survey questionnaire. Twenty five copies each school were distributed. On completion, the questionnaires each school were gathered together and mailed to the researchers for analysis. The teacher in charge of the survey was informed on how to conduct this procedure. Before the survey questionnaire was sent out, the teacher in charge of the survey informed each subject the purpose and procedure of this study. The written informed consent of each subject was obtained.

Before the formal survey, the pretest was carried out in 6 colleges and universities in Chengdu. Eighty copies of survey questionnaires were distributed, and 77 copies were recovered to test and correct the validity and reliability of the scale (notably the adapted scale) in the questionnaire.

### Test of the Validity and Reliability of Scales

Table 1 shows the following:

**TABLE 1:** The quality analysis table for four rating scales.

	Dimension naming	KMO and Bartlett test	Items	VE (%)	Cumulative VE (%)	CR	Cronbach’s α
PTRS	Agreeableness	KMO = 0.88; *P*<0.05	8	24.16	24.16	0.84	0.76
	Conscientiousness Science		6	20.25	44.41	0.87	0.81
	Extroversion		6	16.21	60.62	0.83	0.79
	Neuroticism		7	10.23	70.85	0.79	0.83
	Openness		5	5.59	76.44	0.80	0.78
RRS	Problem cognition	KMO = 0.91; *P*<0.05	8	25.26	25.26	0.86	0.79
	Hope optimism		6	18.35	43.61	0.83	0.83
	Empathy		7	12.37	55.98	0.87	0.80
	Emotion regulation		3	9.58	65.56	0.80	0.87
SSRS			5	–	–	–	–
CTRS	Interactive discussion	KMO = 0.85; *P*<0.05	3	23.27	23.27	0.84	0.80
	Open mind		3	17.78	41.05	0.81	0.87
	Problem-solving		5	13.65	54.70	0.80	0.82
	Multilevel teaching		3	10.26	64.96	0.85	0.81
	Independent learning		6	8.45	73.41	0.83	0.88

**PTRS, the personality trait rating scale; RRS, the resilience rating scale; SSRS, the school support rating scale; CTRS, the creative teaching rating scale; VE, variance explained; CR, composite reliability.**

(1) The personality trait rating scale with 32 items used the original one directly. The critical value CR (all *P*’s < 0.05) of each item in the scale reached a significant level; the exploratory factor analysis (KMO = 0.88; *P* < 0.05, very suitable for factor analysis) showed which could extract the five common factors and the cumulative contribution rate of the five factors reached 76.44%. In terms of reliability, the Cronbach’s α coefficients of the five factors ranged 0.76–0.83, and the overall α coefficient was 0.77. The confirmatory factor analysis showed that the goodness-of-fit index AGFI, CFI, NFI, and IFI were 0.97, 0.94, 0.91, and 0.92, respectively. All of them were greater than 0.90, RMSEA = 0.036 (less than 0.05 fit well); besides, the composite reliability of the five factors (latent variables) was above 0.79, showing the reliability and validity of this scale as good.
(2) The resilience rating scale was an adapted scale. After this 27-item scale was analyzed in terms of critical value CR (all *P*’s < 0.05) and exploratory factor analysis (KMO = 0.91; *P* < 0.05, very suitable for factor analysis), three items were removed; a total of four common factors were extracted, named “problem cognition” (8 items), “hope optimism” (6 items), “empathy” (7 items), and “emotion regulation” (3 items). The cumulative contribution rate was 65.56%. In terms of reliability, the overall Cronbach’s α coefficient was 0.81; after confirmatory factor analysis, the goodness-of-fit indexes AGFI, CFI, NFI, and IFI were 0.95, 0.91, 0.93, and 0.95, respectively, all of which were greater than 0.90. RMSEA = 0.041 (less than 0.05 fit well). In addition, the composite reliability of the four factors (latent variables) was above 0.80, which showed that the scale has good reliability and validity.
(3) The school support rating scale was single-dimensional. The analysis of the scale showed that the critical value CR of the five items reached a significant level; after confirmatory factor analysis, AGFI, CFI, NFI, and IFI were 0.92, 0.93, 0.91, and 0.96, respectively, all of which were greater than 0.90, RMSEA = 0.048 (less than 0.05 fits well). The overall Cronbach’s α was 0.89, which showed that the scale has good reliability and validity.
(4) The creative teaching rating scale also used the original scale completely. After item analysis and exploratory factor analysis (KMO = 0.85; *P* < 0.05, which was very suitable for factor analysis), the five common factors extracted were consistent with the original scale. The cumulative contribution rate was 73.41%. Among them, interactive discussion refers to promoting students’ ability to analyze and think through topic discussion and interaction; open mind refers to maintaining an open mind and flexible adjustment of teaching content, and emphasizing the connection with life to cultivate students’ adaptability; problem-solving refers to passing questions, and metaphors are used to enhance students’ problem-solving knowledge and imagination; multilevel teaching refers to the use of diverse teaching materials or activities to enhance students’ concentration, curiosity, and motivation; and independent learning refers to self-directed learning activities and challenging assignments, encouraging, and improving autonomous learning. After confirmatory factor analysis, the goodness-of-fit index AGFI, CFI, NFI, and IFI were 0.93, 0.96, 0.92, and 0.93, respectively, RMSEA = 0.039. In addition, the composite reliability of the five factors (latent variables) was above 0.80, which showed that the scale has good reliability and validity.

### Data Analysis

SPSS 17.0 and AMOS version 17.0 statistical package analysis software was used to execute the data collected by a questionnaire. Using exploratory (EFA) and confirmatory (CFA) factor analysis methods, and multiple stratified regression methods to process the corresponding data, the significance level of all statistics was set to α = 0.05.

## Results

### Correlation Analysis of Each Dimension of Personality Traits, Resilience, School Support, and Creative Teaching

The correlation analysis of each dimension of personality traits, resilience, school support, and creative teaching in Table 2 shows as in the following:

**TABLE 2:** Correlation coefficient matrix between school support, personality traits, resilience, and creative teaching.

Variables	M ± *SD*	SS	X_1_	X_2_	X_3_	X_4_	X_5_	Z_1_	Z_2_	Z_3_	Z_4_	Y_1_	Y_2_	Y_3_	Y_4_	Y_5_
SS	4.11 ± 0.38	1.00														
X_1_	4.69 ± 0.59	0.12	1.00													
X_2_	4.50 ± 0.47	0.24*	0.70*	1.00												
X_3_	4.22 ± 0.62	0.09	0.65*	0.61*	1.00											
X_4_	3.12 ± 0.49	−0.26*	−0.52*	−0.39*	−0.42*	1.00										
X_5_	4.18 ± 0.55	0.15	0.56*	−0.61*	0.73*	−0.34*	1.00									
Z_1_	4.56 ± 0.71	0.36*	0.65*	0.71*	0.68*	−0.44*	0.63*	1.00								
Z_2_	4.49 ± 0.64	0.37*	0.33*	0.57*	0.50*	0.68*	−0.55*	0.54*	1.00							
Z_3_	4.85 ± 0.78	0.32*	0.78*	0.66*	0.61*	−0.39*	0.58*	0.71*	0.64*	1.00						
Z_4_	4.29 ± 0.61	0.34*	0.51*	0.49*	0.44*	−0.40*	0.42*	0.56*	0.68*	0.62*	1.00					
Y_1_	4.51 ± 0.78	0.35*	0.45*	0.50*	0.57*	−0.19*	0.54*	0.55*	0.46*	0.52*	0.41*	1.00				
Y_2_	4.95 ± 0.69	0.23*	0.59*	0.51*	0.44*	−0.32*	0.44*	0.58*	0.47*	0.62*	0.39*	0.64	1.00			
Y_3_	4.69 ± 0.78	0.29*	0.56*	0.53*	0.52*	−0.24*	0.56*	0.62*	0.55*	0.60*	0.51*	0.77	0.77	1.00		
Y_4_	4.58 ± 0.57	0.33*	0.52*	0.48*	0.55*	−0.27*	0.59*	0.54*	0.51*	0.59*	0.48*	0.75	0.65	0.77	1.00	
Y_5_	4.30 ± 0.66	0.38*	0.43*	0.45*	0.59*	−0.21*	0.51*	0.50*	0.52*	0.51*	0.50*	0.81	0.60	0.71	0.78	1.00

M ± SD, mean ± standard deviation; SS, school support; X_1_, agreeableness; X_2_, conscientiousness; X_3_, extroversion; X_4_, neuroticism; X_5_, openness; Z_1_, problem cognition; Z_2_, optimism; Z_3_, empathy; Z_4_, emotion regulation; Y_1_, interactive discussion; Y_2_, open mind; Y_3_, problem-solving; Y_4_, multilevel teaching; Y_5_, autonomous learning. *p < 0.05.

(1) There were significant positive correlations between school support (SS) and each construct of resilience (i.e., problem cognition, hope optimism, empathy, and emotional regulation, respectively), as well as significant positive correlations between SS and each construct of creative teachings (i.e., interactive discussion, open-mindedness, problem solving, multiple learning, and autonomous learning, respectively); there were significant positive correlations between each construct of resilience and creative teachings.
(2) Each construct of personality traits (agreeableness, conscientiousness, extroversion, neuroticism, and openness) was significantly correlated with resilience; personality traits were significantly correlated with creative teaching.

On combining the above two aspects (1) and (2), it can be affirmed that there is a close relationship between school support, resilience, and creative teaching, personality traits, resilience, and creative teaching, so it is suitable for multilevel analysis.

### Analysis of the Influence of Personality Traits and Resilience on Creative Teaching

The analysis of the influence of personality traits and resilience on creative teaching in Table 3 shows:

**TABLE 3:** Multiple linear regression analysis of personality traits and resilience on creative teaching.

	Dependent variables	Personality traits					Resilience				Statistical tests		
				
		X_1_	X_2_	X_3_	X_4_	X_5_	Z_1_	Z_2_	Z_3_	Z_4_	R	*R*^2^	*F*
CT	Y_1_ (β)	0.14	0.15	0.31*	−0.19*	0.26*					0.685	0.4692	56.21*
	Y_2_ (β)	0.56*	0.07	0.06	–0.09	0.16*					0.662	0.4382	35.47*
	Y_3_ (β)	0.33*	0.11	0.14*	–0.08	0.27*					0.678	0.4596	30.45*
	Y_4_ (β)	0.29*	0.06	0.17*	−0.15*	0.32*					0.694	0.4816	48.12*
	Y_5_ (β)	0.12	0.08	0.32*	−0.14*	0.25*					0.643	0.4134	29.26*
Re	Z_1_ (β)	0.16*	0.34*	0.23*	–0.06	0.09					0.589	0.3469	30.15*
	Z_2_ (β)	0.14	0.05	0.48*	−0.49*	0.26*					0.605	0.3660	35.21*
	Z_3_ (β)	0.71*	0.07	0.16*	−0.11*	0.13*					0.624	0.3893	28.77*
	Z_4_ (β)	0.40*	0.09	0.16*	−0.25*	0.08					0.551	0.3036	21.23*
CT	Y_11_ (β)	–0.07	0.05	0.20*	−0.18*	0.24*	0.17*	0.07	0.12	0.11*	0.795	0.5487	45.29*
	Y_22_ (β)	0.27*	0.04	0.06	0.07	0.15*	0.15*	0.03	0.34*	0.06	0.694	0.5205	45.15*
	Y_33_ (β)	0.13	0.03	0.04	–0.06	0.18*	0.24*	0.07	0.15*	0.04	0.684	0.5147	38.15*
	Y_44_ (β)	0.09	0.03	0.07	0.18*	0.24*	0.11	0.14*	0.21*	0.06	0.752	0.5547	51.26*
	Y_55_ (β)	0.07	0.01	0.24*	0.15*	0.19*	0.14*	0.04	0.16*	0.15*	0.691	0.4734	36.17*

CT, creative teaching; Re, resilience; X_1_, agreeableness; X_2_, conscientiousness; X_3_, extroversion; X_4_, neuroticism; X_5_, openness; Z_1_, problem cognition; Z_2_, optimism; Z_3_, empathy; Z_4_, emotion regulation; Y_1_, interactive discussion; Y_2_, open mind; Y_3_, problem-solving; Y_4_, multilevel teaching; Y_5_, autonomous learning; β, regression coefficients. *P < 0.05.

(1) From the direct influence of personality traits on creative teaching: Regression equation 1 (i.e., Y_1_, interactive discussion) was statistically significant (*F* = 56.21, *P* < 0.01). Among the five personality trait variables, the three traits extroversion, neuroticism, and openness reached significance levels. Regression equation 2 (i.e., Y_2_, openness) was statistically significant (*F* = 35.47, *P* < 0.01); among the five personality traits, the two traits agreeableness and openness reached significant levels. Regression equation 3 (i.e., Y_3_, problem solving) was statistically significant (*F* = 30.45, *P* < 0.01); among the five personality traits, the three traits agreeableness, extroversion, and openness reached a significant level. Regression equation 4 (i.e., Y_4_, multiple teaching) was statistically significant (*F* = 48.12, *P* < 0.01); among the five personality traits, the agreeableness, extroversion, neuroticism, and openness reached significance levels.
(2) From the influence of personality traits on resilience. The resilience regression equation Z_1_ (problem cognition) reached a significance level (*F* = 30.15, *P* < 0.01), and the agreeableness, conscientiousness, and extroversion among the five personality traits reached significance levels; regression equation Z_2_ (hopefully optimistic) reached a significant level (*F* = 35.21, *P* < 0.01), and the agreeableness, extroversion, neuroticism, and openness of the five personality traits reached significance levels; regression equation Z_3_ (empathy) reached a significance level (*F* = 28.77, *P* < 0.01), and the agreeableness, extroversion, neuroticism, and openness among the five personality traits reached significance levels; regression equation Z_4_ (emotional regulation) reached a significance level (*F* = 21.23, *P* < 0.01), and the agreeableness and extroversion among the five personality traits, as well as the neuroticism, reached a significance level.
(3) When both the four dimensions of resilience (Z_1_–Z_4_) and the five dimensions of personality traits (X_1_–X_5_) were entered into the regression model, regression equation Y_11_ (interactive discussion) showed that “problem cognition” and “emotional regulation” in resilience had significant predictive power for interactive discussions, and “extroversion” could significantly affect “problem cognition” and “emotion regulation,” so it can be inferred that “extroversion” produced mediation. Regression equation Y_22_ (openness) shows that “problem cognition” and “empathy” in resilience had a significant predictive power for “openness,” while “agreeableness” could significantly affect “problem cognition” and “empathy,”; it can be inferred that “agreeableness” had some intermediary influence on “openness” via “problem cognition” and “empathy.” Regression equation Y_33_ (problem solving) shows that “problem cognition” and “empathy” in resilience had significant predictive power, and “agreeableness” and “extroversion” through “problem cognition” and “empathy” had a full mediation effect, because the original regression coefficient has been reduced from significant levels to insignificance levels, and “openness” had a partial intermediary via “problem recognition” and “empathy.” Regression equation Y_44_ (multiple teaching) showed that the “hope optimism” and “empathy” in resilience had a significant predictive power for multilevel teaching, of which “agreeableness” and “extroversion” had a full intermediary via “hope optimism” and “empathy,” because the original regression coefficients were significant), but now become insignificant; “openness” had a partial intermediary via “hope optimism” and “empathy,” because the original regression coefficient was 0.32^∗^, which was still significant but dropped to 0.24^∗^. The regression equation Y_55_ (autonomous learning) suggested that the “problem cognition,” “empathy,” and “emotional regulation” in resilience had an intermediary effect, and “extroversion” and “openness” had some intermediaries via “problem cognition” and “empathy,” because the original regression coefficients were 0.32^∗^ and 0.25^∗^, which was still significant but dropped to 0.24^∗^, 0.19^∗^; extroversion also had some intermediaries via “emotion regulation,” because the original regression coefficient was 0.32^∗^, which was still significant, but to 0.24^∗^.

### Analysis of the Influence of School Support and Resilience on Creative Teaching

The analysis of the influence of school support and resilience on creative teaching in Table 4 shows:

**TABLE 4:** Multivariance linear regression analysis of school support and resilience on creative teaching.

	β of creative teaching					β of resilience				β of creative teaching				
			
	Y_1_	Y_2_	Y_3_	Y_4_	Y_5_	Z_1_	Z_2_	Z_3_	Z_4_	Y_1_	Y_2_	Y_3_	Y_4_	Y_5_
SS	0.44*	0.35*	0.35*	0.30*	0.42*	0.39*	0.31*	0.37*	0.33*	0.25*0.07	0.43*	0.19*	0.19*	0.45*
Z_1_										0.30*	0.04	0.33*	0.21*	0.10
Z_2_										0.13	0.03	0.04	0.14	0.17*
Z_3_										0.24*	0.03	0.25*	0.31*	0.27*
Z_4_										0.07	0.01	0.11	0.13	0.06
R	0.68	0.85	0.86	0.56	0.72	0.52	0.64	0.67	0.47	0.75	0.89	0.90	0.66	0.79
R^2^	0.4622%	0.723	0.740	0.314	0.518	0.270	0.410	0.449	0.221	0.563	0.792	0.810	0.436	0.624
F	26.3*	17.5*	22.6*	18.4*	15.7*	21.4*	30.2*	18.4*	26.3*	31.2*	41.7*	22.9*	30.7*	41.4*

*P < 0.05. SS school support; X_1_, agreeableness; X_2_, conscientiousness; X_3_, extroversion; X_4_, neuroticism; X_5_, openness; Z_1_, problem cognition; Z_2_, optimism; Z_3_, empathy; Z_4_, emotion regulation; Y_1_, interactive discussion; Y_2_, open mind; Y_3_, problem-solving; Y_4_, multilevel teaching; Y_5_, autonomous learning.

(1) Taking the school support (SS) as the independent variable, and the five dimensions of creative teaching as the dependent variables separately for regression analysis, it was found that all the five regression equations reached significance levels, indicating that school support (SS) significantly restricted the level of teachers’ creative teaching; for the open-mindedness (Y_2_) and problem-solving (Y_3_) in creative teaching, group-level intervention (school support) was significantly more effective in the five personality traits and resilience than the individual level.
(2) Taking school support (SS) as the independent variable and the four dimensions of resilience as the dependent variables for regression analysis, it was found that the four regression equations all reached a significance level, including problem cognition, hope optimism, empathy, or emotional regulation.
(3) Taking the five dimensions of teacher creativity as the dependent variables and school support (SS) as well as the four dimensions of teacher resilience as independent variables for regression analysis, it was found that the coefficient β corresponding to school support (SS) in equation Y_1_ (interactive discussion) was 0.44^∗^, decreased to 0.25^∗^, and reached a significant level, indicating that the school support of the mediating effect on interactive discussions through teacher resilience; in the four dimensions of resilience in Equation Y_1_, only problem cognition and empathy reached a significant level (β = 0.30^∗∗^, and 0.24 ^∗∗^), indicating the intermediary effect of SS → Z_1_ → Y, SS → Z_3_ → Y of the school support established. The coefficient β corresponding to the school support (SS) in equation Y_2_ (open-mindedness) was increased from 0.35^∗^ to 0.43^∗^, so it had no intermediary effect. The coefficient β corresponding to the school support (SS) in equation Y_3_ (problem solving) has decreased from the original 0.35^∗^ to 0.19^∗^ and has reached a significant level, indicating that the school support had a partial mediation effect on problem solving through teacher resilience. Among the four dimensions of resilience, only problem cognition and empathy had significant levels (β = 0.33^∗^, 0.25 ^∗^), indicating that the school support of SS → Z_1_ → Y, SS → Z_3_ → Y had indirect effects. The coefficient β corresponding to school support (SS) in equation Y_4_ (multiple teaching) has dropped from 0.30^∗^ to 0.19^∗^, which also reached a significant level, indicating that school support has a partial intermediary effect on multilevel teaching through teacher resilience. Among the four dimensions, only problematic cognition and empathy reached a significant level (β = 0.21^∗^, 0.31^∗^), indicating that the indirect effect of school support of SS → Z_1_ → Y, G → Z_3_ → Y was established. Finally, the coefficient β corresponding to school support (SS) in equation Y_5_ (autonomous learning) increased from 0.42^∗^ to 0.45^∗^, reaching a significant level, indicating that the intermediary effect did not exist.

## Analysis and Discussion

### From the Relationship of Personality Traits to Creative Teaching

This study found that among the five dimensions of personality traits, neuroticism was significantly positively correlated with each dimension of creative teaching, while extroversion, openness, agreeableness, and conscientiousness were significantly negatively correlated. That is, the higher the neuroticism scored, the worse the creative teaching ability tended to be, while the extroversion, openness, agreeableness, and conscientiousness scores were high, and the creative teaching ability was also high. These findings support the views of most previous studies (Chen et al., 2014; Chao et al., 2017). For example, people who were good at creativity often had certain specific personality traits, and teachers who performed well in scientific competitions had many personal traits and abilities that were conducive to creation, such as a wide range of interests, attitudes that were not constrained by prejudice and old methods, and cognitive skills related to creativity (Miron-Spektor and Beenen, 2015; Chernyavskaya and Samoylichenko, 2016). Individuals with high neuroticism were prone to have a sense of inferiority and minded others’ ridicule and accusation, often because they were afraid of their opinions or ideas not being recognized by others, leading to new pressure, which reduced the individual’s willingness to originality teaching (Boyer and Byrnes, 2009). Teachers with high extroversion were good at socializing, and they were lively and talkative and liked to cooperate with others, so they had a higher creative teaching behavior (Huang et al., 2017). Teachers with high openness traits had rich imagination, hobby diversity, strong curiosity, independent thinking, and no prejudice, so people with high openness will tend to have creative teaching (Rodrigues and Rebelo, 2013). People with high agreeableness traits were more courteous, supple, kind, cooperative, easy to trust others, tolerate others, and willing to share their views and impart their knowledge with others, so the willingness to carry out creative teaching was high (Hiedanpää, 2005; Soomro et al., 2016). People with higher scores in conscientiousness were more independent, cautious, self-conscious, and perseverant, so the higher the degree of conscientiousness, the higher the degree of creative teaching (Whaite et al., 2018).

This study found that among the five personality traits, the four dimensions of agreeableness, extroversion, neuroticism, and openness (except for conscientiousness) had varying degrees of influence on the five dimensions of creative teaching, of which openness had the most extensive influence, followed by extroversion, while the influence of agreeableness and neuroticism was less; moreover, the influence of neuroticism was negative. From the determinant coefficient *R*^2^ value of the regression equation, the influence of personality traits on “interactive discussion,” “open-mindedness,” “problem-solving,” “multiple teaching,” and “autonomous learning” was 46.92, 43.82, 45.96, 48.16, and 41.34%, respectively. These results also support some views of some scholars (Rodrigues and Rebelo, 2013; Chernyavskaya and Samoylichenko, 2016) but were inconsistent with other views (Soomro et al., 2016; Whaite et al., 2018). For example, the present study found that “conscientiousness” had no significant effect on the five dimensions of creative teaching, which seemed difficult to obtain an explanation. The role of “conscientiousness” in creative teaching is to hope that their ideas are understood and to communicate with others to present their views for improvement. For physical education teachers, it is likely that they are not good at giving their views, thereby the results were not significant. It seems that this issue needs to be further explored.

### From the Impact of Personality Traits on Resilience

This study found that “agreeableness,” “conscientiousness,” and “extroversion” in positive personality traits were significantly positively correlated with each dimension of the resilience. In contrast, except the negative “neuroticism” in personality traits which was significantly positively correlated with “hope optimism” in resilience, the others were significantly positively correlated with “problem-solving” but negatively correlates with “problem-solving,” “empathy,” and “emotion regulation,” while the expressions of “openness” and “neuroticism” in personality traits were the opposite. Regression analysis further revealed that “agreeableness,” “conscientiousness,” and “extroversion” in personality traits could significantly explain 34.69% of the variation of “problem cognition” in resilience; agreeableness, extroversion, openness, and neuroticism could significantly explain 36.6 and 38.93% of the variant of “hope optimism” and “empathy” in resilience; and agreeableness, extroversion, and neuroticism could significantly explain 30.36% of the variant of the “emotional regulation” in resilience. According to related research reports, positive personality traits were an essential element of positive psychology. They include advantages, positive emotions, optimism, resilience, gratitude, and mental flow. Among them, resilience, advantages, and hope are the most important (Krupić et al., 2016). Hallak et al. (2018) found that personality traits were the most critical factors affecting creativity performance. People with high creativity and high resilience had many common personal traits; individuals with high scientific creativity had more remarkable resilience performance than the average individual. Individuals with resilience were good at handling multiple competitive stimuli and were more able to resist temptation and distinguish false appearances. Therefore, those with better resilience tend to respond more in changing situations, especially in frustration and stress situations and have excellent characteristics such as flexibility, non-rigidity, functional adaptation, and more effective interpersonal interaction (Perry and Karpova, 2017). Therefore, the results of this study were consistent with the views of some other scholars.

### From the Perspective of Resilience as the Intermediary Role of Personality Traits and Creative Teaching

This study found that “extroversion” in personality traits of physical education teachers can have some intermediary effects through “problem cognition” and “emotional regulation” in resilience to affect “interactive discussion” in creative teaching. “Openness” could produce some intermediary effects through “problem cognition” and “empathy” in resilience, while “agreeableness” and “extroversion” in personality traits could be used through “problem cognition” in resilience. “Empathy” had full intermediaries that affect “problem-solving” in creative teaching. “Openness” in personality traits could have partial intermediaries through “hope optimism” and “empathy” in resilience, and “agreeableness” and “extroversion” in personality traits had the full intermediary effects on “multiple teaching” in creative teaching through “hope optimism” and “empathy” in resilience. “Extroversion” and “openness” in personality traits had some intermediary effects through “problem cognition” and “empathy” in resilience, while “extroversion” also had partial intermediary impact on “self-directed learning” in creative teaching via “emotional regulation” in resilience. “Agreeableness” and “openness” in personality traits had some intermediary influence on “open-mindedness” in creative teaching via “problem cognition” and “empathy” in resilience.

The practice of physical education has proven that the creative physical education process is often complicated. It requires teachers to systematically and creatively design teaching programs, to use various appropriate teaching techniques, to change teaching methods, and to arrange teaching activities. Because of the open teaching environment, high frequency of physical activities, and many opportunities for group interaction and cooperation in the process of physical education, the teaching methods and teaching materials used by teachers are more challenging. From the emergence of a creative idea in mind to the output of innovative design, it must often go through countless attempts and experiments. More often, it is necessary to face and adjust the emotion after failure and use other strategies to build a beneficial environment to their creative teaching environment, or find some available support and resources, so that they continue to bet on innovative teaching. However, so far, few scholars can comprehensively explore the relationship between physical qualities, resilience, and the creativity of physical education teachers. This study verified the multi-role and multilevel intermediary adjustment effectiveness of resilience, so it can be considered that physical education teachers with better resilience function may face in changing situations, especially in frustration and stress situations, more flexibility, more inflexibility, better adaptability, and better interpersonal interaction ability. So in creative teaching, despite facing many pressures and setbacks, resilience supports them to face this adversity and reduce the impact of delays.

### From the Correlation Between School Support and Creative Teaching

Chiang and Hsieh (2012) pointed out that after employees perceived organizational support, they would increase employee participation and performance through two ways to achieve organizational goals. One was that the employee would expect that when he strived to help the organization achieve its goals, the organization would provide him with a relevant reward result, and this predicted reward would make the employees think that the organization valued their contribution, so it produced return psychology; the other was to cultivate positive emotional attachment to the organization. In other words, organizational support emphasizes that employees believe that the organization treats them positively. Under the influence of social exchange and psychological contracts, employees will display different work attitudes and behaviors according to their perceived degree of organizational support. In the field of education, when discussing creative teaching strategies, Rivas (2017) found that adequate resources were the key to teachers’ innovative teaching. If excellent teachers receive more support from principals, directors, and other administrative supervisors, they feel that the school encourages teachers to implement creative teaching will inspire them to try creative teaching activities. When discussing issues related to the creative teaching of physical education teachers, Zhang and Xie (2009) believed that there are indeed many factors in the teaching environment that will affect teachers’ creative teaching performance, such as supervisors’ timely encouragement and administrative support for teachers’ creative display in teaching.

As discussed above, the present study equated organizational support with school support and found that school support has a significant influence on the five dimensions of creative teaching (Y_1_–Y_5_), with powers of 46.2, 72.3, 74.0, 31.4, and 51.8%, respectively. Moreover, this influence generally exceeded the importance of teachers’ personality traits on creative teaching (which were 46.92, 43.82, 45.96, 48.16, and 41.34%, respectively), which showed that the findings of this study were similar to those of previous related disciplines, and it also validated the hypothesis of this study that school support has a positive effect on teachers’ creative teaching behavior.

### From the Perspective of Resilience as the Intermediary Effect of School Support and Creative Teaching

This study found that school support, as an independent variable, had a significant influence on the four dimensions of resilience (i.e., problem cognition, hope optimism, empathy, or emotional regulation); its explanatory power amounted to 27.0, 41.0, 44.9, and 22.1%, respectively. On the other hand, the school support had partial mediation effects on “interactive discussion,” “problem-solving,” and “multiple teaching” in creative teaching through the two dimensions of “problem recognition” and “empathy” in resilience. In other words, school support had an impact on creative teaching through the partial resilience of teachers. This result is entirely consistent with the focus in the theory of organizational support and is also compatible with previous research results (Liu et al., 2015; Akgunduz and Sanli, 2017; Wang et al., 2017). That is, if teachers feel the support of the school, they will meet the two aspects problem cognition and empathy in resilience to obtain higher intermediary power, which is consistent with the cognition component of the “reciprocity mechanism” in supporting theory which has an intermediary effect. The present study differs from previous explorations in that there are currently few scholars at home and abroad can explore the resilience of physical education teachers as multiple intermediary variables. Most of them are only dealt with at the individual level (personality traits) and less comprehensively discuss the intermediary effects produced by the joint action of individuals (personal traits) and groups (school factors); this is the most significant contribution of this study compared to previous studies.

Besides, studies in education-related fields have also found that when discussing creative teaching strategies, the availability of resources is the key to teaching innovation. If the principals, directors, and other administrative supervisors can give more support in the teaching process, the teachers will feel that the school will inspire teachers to try creative teaching activities (Sanders, 2004). Some scholars have found that there are indeed many factors that affect original teaching performance in the teaching environment, including supervisors who provide timely encouragement and administrative support for teachers to show creativity in teaching (Chiou, 2002). It can be seen that under the influence of the individual and the environment, the interaction between the individual and the environment still has a non-negligible role. The creative teaching performance of individual teachers is not affected by different factors or group factors alone, but a kind of the product of the interaction between group factors. In other words, personal resilience is likely to interact with school support and affect creative teaching. Therefore, the argument that school support will play a moderating effect between teacher resilience and original teaching performance should be established. That is, school support should have a positive impact on creative teaching behavior. Accordingly, school support has a positive effect on original teaching behavior.

## Conclusion and Recommendations

### Conclusion

(1) Agreeableness has a positive influence on open-mindedness, problem-solving, and multiple teaching. Extroversion has a positive influence on interactive discussion, problem-solving, multiple teaching, and autonomous learning; neuroticism has a negative influence on interactive discussion, multiple teaching, and autonomous learning; openness has a positive influence on interactive discussions, open-mindedness, and problem-solving; while conscientiousness has no power on creative teaching.
(2) Extroversion can produce partial mediation effects on “interactive discussion” through “question cognition” and “emotion regulation.” Openness can produce partial mediation effects, while agreeableness and extroversion can produce completed mediation effects on “problem-solving” through problem cognition” and “empathy.” Openness can produce partial mediation effects while agreeableness and extroversion can produce completed mediation effects through “hope optimism” and “empathy” on “multiple teaching.” Extroversion and openness can produce partial mediation effects through “problem cognition” and “empathy,” and extroversion can also produce partial mediation effects through “emotion regulation” on autonomous learning. Agreeableness and openness have partial mediation effects on “open-mindedness” through “problem cognition” and “empathy.”
(3) School support has a positive influence on various aspects of creative teaching, especially for the two aspects of creative teaching: “open-mindedness” and “problem-solving.” The impact of school support is more significant than the personality on resilience. The influence of open-mindedness is only affected by school support, not affected by personality traits and resilience.
(4) The school support has a direct positive influence on the five dimensions of creative teaching. At the same time, it can also have partial mediation effects on interactive discussion, problem-solving, and multiple teaching in creative teaching through “problem recognition” and “empathy” in resilience. Problem cognition plays the role of multilevel regulating intermediary effect; that is, school support will affect problem-solving and multi-teaching through problem cognition mediator and positively regulate problem-solving and multilevel teaching impact.

### Recommendation

(1) Four personality traits of physical education teachers have a positive impact on their creative teaching. It is recommended that when recruiting teachers or constructing innovative teaching teams, schools should consider the teachers’ qualifications and teaching level and also pay more attention to understand the personality trait of teachers to estimate their role in the future.
(2) The resilience of physical education teachers is an essential intermediary variable. It can be used as an intermediary not only in personality traits and creative teaching but also in school support and innovative teaching, especially the “problem cognition” in resilience which is the most important. Therefore, schools should attach great importance to the development of teachers’ resilience, to improve and enhance teachers’ awareness of “problems cognition,” and to encourage them to face up to setbacks and know how to use appropriate internal and external resources to solve problems.
(3) School support has a positive influence on the five aspects of creative teaching. The school support emphasized by the present study is mainly aimed at whether the school attaches importance to the job achievements, welfare, and contributions of sports teachers. These connotations are more focused on the value of sports teachers and the respect they deserve, and this atmosphere will promote the creative teaching of sports teachers. Performance has a positive impact, and it is reasonable. Therefore, it is recommended that schools highly respect the job value of teachers. In the overall plan of school teaching reform, physical education should be included in the discussion and cooperation to increase the opportunities for physical education teachers to communicate.

## Data Availability Statement

The raw data supporting the conclusions of this article will be made available by the authors, without undue reservation, to any qualified researcher.

## Ethics Statement

Ethical review and approval was not required for the study on human participants in accordance with the local legislation and institutional requirements. The patients/participants provided their written informed consent to participate in this study.

## Author Contributions

QD and JC designed the study and carried out the survey. QD and BZ analyzed and processed the data. QD wrote the first draft. All authors revised the work.

## Conflict of Interest

The authors declare that the research was conducted in the absence of any commercial or financial relationships that could be construed as a potential conflict of interest.
